# White matter microstructure relates to motor outcomes in myotonic dystrophy type 1 independently of disease duration and genetic burden

**DOI:** 10.1038/s41598-021-84520-2

**Published:** 2021-03-01

**Authors:** Timothy R. Koscik, Ellen van der Plas, Laurie Gutmann, Sarah A. Cumming, Darren G. Monckton, Vincent Magnotta, Richard K. Shields, Peggy C. Nopoulos

**Affiliations:** 1 Department of Psychiatry, Carver College of Medicine, University of Iowa, 200 Hawkins Drive, Iowa City, IA 52242 USA; 2 Department of Neurology, Carver College of Medicine, University of Iowa, Iowa City, USA; 3 Institute of Molecular, Cell and Systems Biology, University of Glasgow, Glasgow, Scotland; 4 Department of Radiology, Carver College of Medicine, University of Iowa, Iowa City, USA; 5 Department of Physical Therapy and Rehabilitation Science, Carver College of Medicine, University of Iowa, Iowa City, USA; 6 Department of Pediatrics, Carver College of Medicine, University of Iowa, Iowa City, USA

**Keywords:** Diseases of the nervous system, Motor control, Neuroscience, Biomarkers, Diseases, Neurology

## Abstract

Deficits in white matter (WM) integrity and motor symptoms are among the most robust and reproducible features of myotonic dystrophy type 1 (DM1). In the present study, we investigate whether WM integrity, obtained from diffusion-weighted MRI, corresponds to quantifiable motor outcomes (*e.g.*, fine motor skills and grip strength) and patient-reported, subjective motor deficits. Critically, we explore these relationships in the context of other potentially causative variables, including: disease duration, elapsed time since motor symptom onset; and genetic burden, the number of excessive CTG repeats causing DM1. We found that fractional anisotropy (a measure of WM integrity) throughout the cerebrum was the strongest predictor of grip strength independently of disease duration and genetic burden, while radial diffusivity predicted fine motor skill (peg board performance). Axial diffusivity did not predict motor outcomes. Our results are consistent with the notion that systemic degradation of WM in DM1 mediates the relationship between DM1 progression and genetic burden with motor outcomes of the disease. Our results suggest that tracking changes in WM integrity over time may be a valuable biomarker for tracking therapeutic interventions, such as future gene therapies, for DM1.

## Introduction

Myotonic dystrophy type 1 (DM1) is a trinucleotide repeat disorder, classically characterized by prolonged muscle contractions (myotonia), progressive muscle wasting, and weakness^1^. Histopathology and molecular studies of DM1 show that the neuromuscular dysfunction is due to a primary abnormality in muscle function. The transcribed mutant *DMPK* mRNA is toxic and leads to a ‘spliceopathy’ resulting in shifts to multiple alterations in proteins involved with skeletal muscle function in DM1^2^. However, it is also well established that the brain is significantly affected in DM1^3^. Although most of the CNS findings have been focused on linking the brain findings to cognitive and behavioral issues, the extent that brain abnormalities may play a role in neuromuscular dysfunction in DM1 is less well established.

Microstructural white matter (WM) pathology in the central nervous system is one of the most robust and reproducible CNS observations in DM1^4^. Fractional Anisotropy (FA), is obtained with diffusion-weighted magnetic resonance imaging, and is an index of non-uniform movement of water molecules ranging from 0 (unrestricted diffusion as in cerebrospinal fluid) to 1 (directionally restricted diffusion as in well-formed white matter fiber bundles). In DM1, FA has been shown to be significantly reduced throughout the cerebral cortex, with little regional specificity^3,5–14^. Statistically significant and widespread FA decrements are seen in samples as small as nine and ten subjects illustrating the large effect size of FA reduction in DM1^7,11^. Several of these studies have shown correlations between FA and muscle impairment as defined by clinical scales such as the Muscle Impairment Rating Scale (MIRS)^3,7,13^, and other clinical ratings of motor function^9,12^. Two studies have shown strong correlations between FA and quantitative measures of motor function such as hand grip or Purdue Pegboard task^12,14^, suggesting a direct relationship between CNS brain dysfunction and objective measures of motor dysfunction.

In addition to objective measures of motor dysfunction, patient-reported, subjective measures of symptoms play an important role in disease management and treatment for patients with DM1. The Myotonic Dystrophy Health Index (MDHI) is a disease-specific, patient-reported outcome measure for DM1^15^. It is composed of 114 items broken down into 16 individual subscales that collectively measure multi-factorial patient-reported burden of disease. Subjective changes of deterioration, or more importantly, ratings that indicate subjective improvement may be important measures in the context of clinical trials aimed at DM1. It has not been evaluated whether subjective measures of muscle or motor dysfunction also are related to measures of brain pathology.

A major limitation of human brain imaging studies and motor dysfunction studies is that relationships are correlative and therefore do not represent ‘true’ mechanistic relationships – correlations do not prove causality. In evaluating how changes in WM microstructure may drive motor dysfunction, there needs to be careful consideration of other confounding factors such as disease duration and CTG repeat length. In a progressive neurodegenerative disorder such as DM1, muscle symptoms worsen over time. FA has also been shown to be directly correlated with disease duration^12^. Therefore, a correlation between FA and motor dysfunction may be due to temporal coordination of independent disease processes rather than any mechanistic or causal relationship. As with disease duration, the genetic burden of longer CTG repeats may result in spurious correlations between measures of central and peripheral disease symptoms. Both brain FA^13^ and muscle dysfunction^16^ have been shown to be directly related to CTG repeats, suggesting underlying mechanisms links. CTG repeat length and disease duration are related, but independent phenomena. Disease progression may start earlier with longer CTG repeats, while disease manifestation becomes worse over time regardless of at what age it started. Accordingly, no previous report has adjusted for disease duration and CTG repeat length when correlating cross-sectional changes in FA and muscle function.

The present study has three aims: (1) to replicate and extend prior observations of WM degradation in a larger sample of DM1 participants compared to a sample of healthy adults; (2) to replicate and extend prior observations of motor impairment in DM1 by using quantitative measures of motor function as well as subjective measures of perceived motor impairments; and (3) explore the relationships between WM degradation and motor impairments, while controlling for CTG repeat number and DM1 duration.

## Results

Sample characteristics are summarized in Table 1. Briefly, the sample included 69 unaffected individuals and 50 individuals with a positive genetic diagnosis of DM1. There were no significant differences between groups in terms of distribution of sex or mean age. There were 2 individuals with DM1 and 3 unaffected individuals who reported prior diagnosis of Attention Deficit/Hyperactivity disorder (ADHD), but this was unrelated to all of the motor outcomes we measured (all *p*’s > 0.15) except for potentially self reports of myotonia, however the small number of individuals reporting ADHD precludes testing the possibility that ADHD is a contributing factor. Neuropsychological variables are summarized in Table 2. DM1 and unaffected individuals differed on several measures, where individuals with DM1 had higher depression (t(63.07) = − 6.32, CI = − 7.38 to − 3.84, *p* = 2.9e−08), anxiety (t(72.47) = − 4.62, CI = −7.14 to  − 2.84, *p* = 1.6e−05), apathy (t(83.51) = −4.09, CI = −7.44 to − 2.57, *p* = 9.9e−05), and lower full-scale IQ scores (t(105.85) = 4.07, CI = 4.69–13.59, *p* = 9.1e−05), visuospatial memory (t(102.74) = 1.97, CI = − 0.02 to 7.49, *p* = 0.051), word association (t(107.97) = 2.19, CI = 0.40–8.09, *p* = 0.031), trail-making (t(84.76) = − 4.03, CI = − 24.62 to − 8.34, *p* = 0.00012), judgement of line orientation (t(96.20) = 2.42, CI = 0.30–3.05, *p* = 0.018), and Wisconsin card sorting performance (t(71.91) = 3.07, CI = 0.31–1.46, *p* = 0.003). As follow-up analyses, we explored whether these cognitive variables were related to motor outcomes and whether these potential relationships were mediated by white matter integrity.

**Table 1:** Sample characteristics.

	N	Sex Men/Women	Age*	Education*	ePAL**	Disease duration*	MIRS***
Healthy Adults	69	25/44	43.6 (12.9)	16.0 (2.06)	13 (5, 43)	–	–
DM1	50	16/34^a^	46.2 (11.6)^b^	15.8 (2.12)^c^	131 (55, 501)^d^	8.88 (7.90)	13:26:9:2

*in years, mean (SD); **Number of CTG repeats in the longest allele, median (minimum, maximum); ***count per rating, 1:2:3:4; ^a^Χ^2^ = 0.08, *p* = 0.776; ^b^β = 2.68, t = 1.17, *p* = 0.246; ^c^β = − 0.13, t = − 0.34, *p* = 0.736; ^d^Χ^2^ = 85.35, *p* ~ 0.

**Table 2:** Neuropsychology.

	Unaffected	DM1	Group differences
Beck depression Inventory—total	1.66 (2.30)	7.26 (6.14)	t(63.07) = − 6.32, CI = − 7.38 to− 3.84, *p* = 2.9e−08
Beck anxiety inventory—total	2.20 (3.67)	7.19 (7.18)	t(72.47) = − 4.62, CI = − 7.14 to − 2.84, *p* = 1.6e−05
Apathy evaluation scale—self-rating	24.26 (5.00)	29.26 (7.78)	t(83.51) = − 4.09, CI = − 7.44 to− 2.57, *p* = 9.9e−05
WAIS4 full-scale IQ	112.97 (11.63)	103.83 (12.85)	t(105.85) = 4.07, CI = 4.69–13.59, *p* = 9.1e−05
List learning—long-delay, list A, z-score	0.59 (0.68)	0.77 (0.77)	t(104.02) = − 1.36, CI = − 0.45 to 0.08, *p* = 0.18
Rey–Osterrieth complex figure test—delayed-recall raw	15.85 (5.43)	14.54 (5.59)	t(103.80) = 1.28, CI = − 0.72 to 3.34, *p* = 0.2
Brief visuospatial memory test—delay, t-score	52.17 (9.95)	48.44 (10.42)	t(102.74) = 1.97, CI = − 0.02 to 7.49, *p* = 0.051
Controlled oral word association test—total raw	44.71 (10.64)	40.46 (10.23)	t(107.97) = 2.19, CI = 0.40–8.09, *p* = 0.031
Trail making test—B, time	52.64 (17.00)	69.12 (25.86)	t(84.76) = − 4.03, CI = − 24.62 to − 8.34, *p* = 0.00012
Animal naming—total raw	24.16 (5.51)	22.50 (4.04)	t(118.92) = 1.91, CI = − 0.06 to 3.38, *p* = 0.058
Judgement of line orientation—raw	26.17 (3.43)	24.50 (3.94)	t(96.20) = 2.42, CI = 0.30–3.05, *p* = 0.018
Bender-gestalt test—total, scaled	117.76 (8.76)	115.66 (10.74)	t(84.99) = 1.11, CI = − 1.65 to 5.84, *p* = 0.27
Wisconsin card sorting test—categories completed	5.71 (0.99)	4.83 (1.89)	t(71.91) = 3.07, CI = 0.31–1.46, * p* = 0.003
Benton faces—total score	46.93 (3.76)	46.55 (4.37)	t(102.49) = 0.51, CI = − 1.11 to 1.87,* p* = 0.61

### Muscle/motor outcomes

We compared muscle/motor outcomes in healthy adults and individuals affected by DM1 using linear mixed effects (LME) models, including family as a random effect and fixed effects of group, age, and sex (p’s corrected for False Discovery Rate, R^2^ represents marginal variance explained or the variance explained by fixed effects only). DM1 is associated with objective, quantitative decline in motor performance as well as subjective ratings of motor impairments. Individuals affected by DM1 were significantly impaired relative to healthy adults on all quantitative muscle/motor outcomes, including peg board (β = 9.08, CI_95%_ = 3.69–14.5, t_(94.81)_ = 3.31,p_FDR_ = 0.00456, R^2^ = 0.0869), finger tapping (β = − 9.52, CI_95%_ = − 12.8 to − 6.28, t_(110.21)_ = − 5.71, p_FDR_ = 3.35 × 10^–7^, R^2^ = 0.222), grip strength (β = − 10.5, CI_95%_ = − 14.5 to − 6.61, t_(107.33)_ = − 5.25, p_FDR_ = 1.82 × 10^–6^, R^2^ = 0.346). DM1-affected individuals, likewise, reported significant burden of myotonia (β = 18.8, CI_95%_ = 12.4–25.1, t_(104.54)_ = 5.76, p_FDR_ = 6 × 10^–7^, R^2^ = 0.234), mobility (β = 14.4, CI_95%_ = 8.42–20.4, t_(103.64)_ = 4.7, p_FDR_ = 5.59 × 10^–5^, R^2^ = 0.168), upper extremity function (β = 17.1, CI_95%_ = 11.7–22.5, t_(103.59)_ = 6.16, p_FDR_ = 9.62 × 10^–8^, R^2^ = 0.259), and swallowing (β = 14.6, CI_95%_ = 8.67–20.5, t_(101.83)_ = 4.85, p_FDR_ = 3.16 × 10^–5^, R^2^ = 0.178).

### WM microstructure

**Figure 1:**
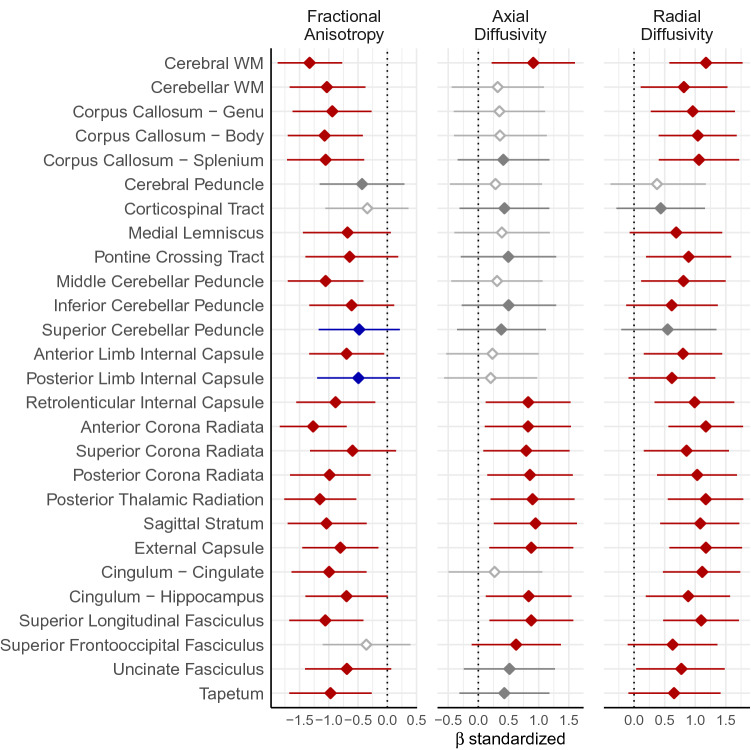
Group differences in WM microstructure measures. Fractional anisotropy values (left panel) were lower in DM1 relative to healthy adults on most regions evaluated (colored estimates), indicating reduced WM integrity. Axial and radial diffusivity (middle and right panels, respectively) were higher in DM1 relative to healthy adults, indicating decreased directional restriction of water molecules in DM1-affected WM. Red indicates volumes of interest (VOIs) where FDR-corrected *p* < 0.05, blue indicates FDR-corrected *p* < 0.1, gray indicates uncorrected *p* < 0.05, and white indicates uncorrected *p* > 0.05. Error bars represent the 99.99% confidence limits, which approximates the FDR-corrected *p* = 0.0081. Data were plotted using R (version 3.5.0, https://www.r-project.org/)^25^.

**Figure 2:**
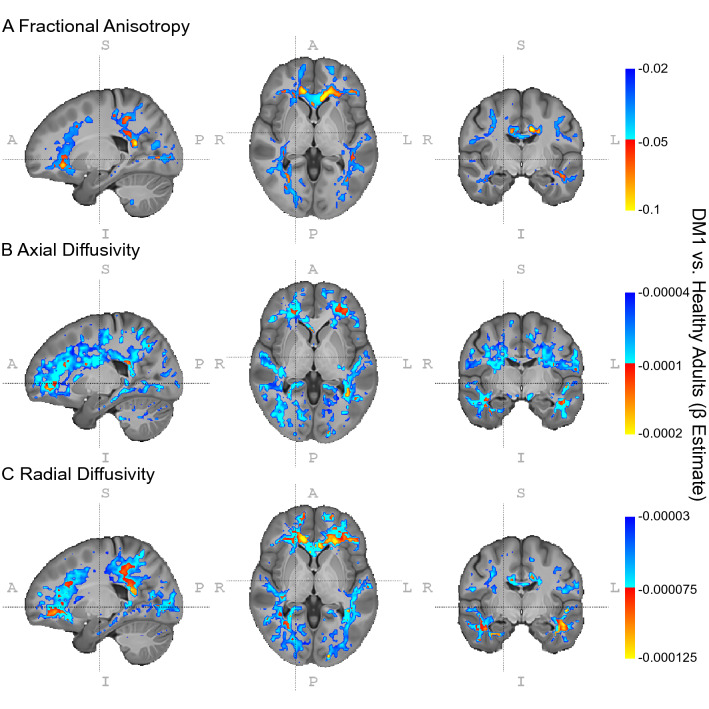
Voxelwise Group Differences in WM microstructure in DM1. WM microstructure in DM1 relative to healthy adults is characterized by decreased fractional anisotropy (panel **A**), and increased axial (panel **B**) and radial diffusivity (panel **C**). This decreased WM integrity in DM1 appears to be systemic rather than regionally specific, though the size of effects varies. Cold colors indicate beta estimates for the group comparison in LME models thresholded at p_uncorrected_ < 0.05; hot colors indicate estimates thresholded at p_uncorrected_ < 0.01. Neuroimages were created using ITK-SNAP (version 3.8.0, http://www.itksnap.org/)^26^. Inkscape (version 0.1, https://inkscape.org/) was used to add labels and color bars to the figure.

Individuals affected by DM1 had significantly degraded WM microstructure, revealed by LME models predicting WM measures with the random effect of family and fixed effects of group, age, and sex. WM microstructure was impacted by DM1 in a systemic rather than regional fashion (see Figs. 1, 2 and Tables 3, 4, 5 for full results); most regions exhibited a decrease in fractional anisotropy (*e.g.*, Cerebral WM FA: β = − 0.0359, CI_95%_ = − 0.0431 to − 0.0285, t_(105.69)_ = − 9.69, p_FDR_ = 1.17 × ^−14^, R^2^ = 0.468), axial diffusivity (*e.g.*, Cerebral WM AD: β = 5.56 × 10^–5^, CI_95%_ = 3.49 × 10^–5^–7.62 × 10^–5^, t_(102.16)_ = 5.22, p_FDR_ = 3.8 × 10^–5^, R^2^ = 0.202), and radial diffusivity (*e.g.*, Cerebral WM RD: β = 6.19 × 10^–5^, CI_95%_ = 4.63 × 10^–5^–7.73 × 10^–5^, t_(108.27)_ = 7.84, p_FDR_ = 1.38e−10, R^2^ = 0.355). Axial diffusivity tended to be less affected by DM1 than radial diffusivity, as shown by more widespread significant differences in RD than AD, *e.g.*, in the corpus callosum, middle cerebellar peduncle, anterior and posterior internal capsule, cingulate cingulum, superior frontooccipital fasciculus, and uncinate fasciculus.

**Table 3:** Group differences in fractional anisotropy.

	β	t(df)	p_uncorrected_	p_FDR_	CI_99.99%_	R^2^
Cerebral WM	− 0.0359	− 9.69 (105.69)	2.89 × 10^–16^	3.9 × 10^–15^	− 0.0431 to − 0.0285	0.468
Cerebellar WM	− 0.0279	− 6.43 (88.30)	6.32 × 10^–9^	8.53 × 10^–8^	− 0.0363 to − 0.0192	0.272
Corpus Callosum—genu	− 0.0695	− 5.54 (93.68)	2.77 × 10^–7^	3.75 × 10^–6^	− 0.0938 to − 0.0451	0.216
Corpus callosum—body	− 0.0584	− 6.75 (90.36)	1.35 × 10^–9^	1.83 × 10^–8^	− 0.0753 to − 0.0411	0.292
Corpus callosum—splenium	− 0.0575	− 6.34 (99.79)	6.88 × 10^–9^	9.29 × 10^–8^	− 0.0752 to − 0.0399	0.269
Cerebral peduncle	− 0.0203	− 2.36 (109.00)	0.0203	0.183	− 0.037 to − 0.00357	0.067
Corticospinal tract	− 0.0161	− 1.92 (81.84)	0.0583	0.524	− 0.0326 to 0.000232	0.145
Medial lemniscus	− 0.0399	− 3.68 (90.87)	0.000391	0.00528	− 0.0618 to − 0.0181	0.112
Pontine crossing tract	− 0.0323	− 3.46 (108.97)	0.000767	0.0104	− 0.0511 to − 0.0121	0.099
Middle cerebellar peduncle	− 0.0332	− 6.45 (109.00)	3.2 × 10^–9^	4.32 × 10^–8^	− 0.0432 to − 0.0232	0.271
Inferior cerebellar peduncle	− 0.0245	− 3.33 (105.86)	0.0012	0.0162	− 0.0389 to − 0.0102	0.094
Superior cerebellar peduncle	− 0.0259	− 2.74 (109.00)	0.00725	0.0653	− 0.0456 to − 0.00751	0.117
Anterior limb internal capsule	− 0.0245	− 4.3 (109.00)	3.68 × 10^–5^	0.000331	− 0.0356 to − 0.0134	0.194
Posterior limb internal capsule	− 0.0164	− 2.77 (108.50)	0.00659	0.0593	− 0.0279 to − 0.00482	0.092
Retrolenticular internal capsule	− 0.0269	− 5.22 (104.45)	9.03 × 10^–7^	1.22 × 10^–5^	− 0.037 to − 0.0168	0.203
Anterior corona radiata	− 0.0502	− 8.83 (107.34)	2.23 × 10^–14^	3.01 × 10^–13^	− 0.0612 to − 0.039	0.421
Superior corona radiata	− 0.0174	− 3.34 (103.34)	0.00115	0.0134	− 0.0279 to − 0.00674	0.094
Posterior corona radiata	− 0.0389	− 5.91 (105.32)	4.33 × 10^–8^	5.84 × 10^–7^	− 0.0521 to − 0.0255	0.246
Posterior thalamic radiation	− 0.0497	− 7.5 (95.66)	3.23 × 10^–11^	4.35 × 10^–10^	− 0.0626 to − 0.0367	0.341
Sagittal stratum	− 0.0426	− 6.26 (106.23)	8.32 × 10^–9^	1.12 × 10^–7^	− 0.056 to − 0.0289	0.268
External capsule	− 0.0221	− 4.88 (109.00)	3.68 × 10^–6^	4.96 × 10^–5^	− 0.0309 to − 0.0133	0.176
Cingulum—cingulate	− 0.0635	− 6.13 (109.00)	1.43 × 10^–8^	1.93 × 10^–7^	− 0.0837 to − 0.0434	0.252
Cingulum—hippocampus	− 0.0233	− 3.92 (109.00)	0.000157	0.00212	− 0.0348 to − 0.0117	0.121
Superior longitudinal fasc	− 0.0349	− 6.91 (104.66)	3.99 × 10^–10^	5.38 × 10^–9^	− 0.045 to − 0.0245	0.309
Superior frontooccipital fasc	− 0.0133	− 1.9 (102.07)	0.0597	0.537	− 0.0269 to 0.000401	0.051
Uncinate fasc	− 0.0399	− 3.91 (96.05)	0.000169	0.00228	− 0.0607 to − 0.0186	0.125
Tapetum	− 0.0521	− 5.47 (108.97)	2.89 × 10^–7^	3.91 × 10^–6^	− 0.0708 to − 0.0336	0.216

**Table 4:** Group differences in axial diffusivity.

	β	t(df)	p_uncorrected_	p_FDR_	CI_99.99%_	R^2^
Cerebral WM	5.56 × 10^–5^	5.22 (102.16)	9.39 × 10^–7^	1.27 × 10^–5^	3.49 × 10^–5^–7.62 × 10^–5^	0.202
Cerebellar WM	2.02 × 10^–5^	1.67 (101.88)	0.0989	0.89	− 3.35 × 10^–6^ to 4.38 × 10^–5^	0.0258
Corpus callosum—genu	4.09 × 10^–5^	1.83 (108.68)	0.07	0.63	− 2.48 × 10^–6^ to 8.44 × 10^–5^	0.0423
Corpus callosum—body	3.21 × 10^–5^	1.86 (102.09)	0.0651	0.586	− 1.49 × 10^–6^ to 6.6 × 10^–5^	0.0371
Corpus callosum—splenium	3.86 × 10^–5^	2.16 (106.00)	0.0327	0.424	3.91 × 10^–6^–7.35 × 10^–5^	0.0422
Cerebral peduncle	2.92 × 10^–5^	1.5 (100.95)	0.138	1	− 9.01 × 10^–6^ to 6.78 × 10^–5^	0.0579
Corticospinal tract	5.03 × 10^–5^	2.3 (109.00)	0.0231	0.312	7.91 × 10^–6^–9.27 × 10^–5^	0.0454
Medial lemniscus	6.85 × 10^–5^	1.98 (105.87)	0.0508	0.686	5.51 × 10^–7^–0.000137	0.0353
Pontine crossing tract	6.46 × 10^–5^	2.58 (107.20)	0.0113	0.152	1.42 × 10^–5^–0.000116	0.0584
Middle cerebellar peduncle	2.59 × 10^–5^	1.63 (93.97)	0.106	0.954	− 4.98 × 10^–6^ to 5.67 × 10^–5^	0.031
Inferior cerebellar peduncle	4.99 × 10^–5^	2.58 (108.01)	0.0113	0.152	1.18 × 10^–5^–8.85 × 10^–5^	0.0583
Superior cerebellar peduncle	6.88 × 10^–5^	2.05 (100.98)	0.0429	0.386	3.64 × 10^–6^–0.000134	0.0615
Anterior Limb internal capsule	1.29 × 10^–5^	1.2 (108.20)	0.231	1	− 7.88 × 10^–6^ to 3.37 × 10^–5^	0.0421
Posterior limb internal capsule	1.33 × 10^–5^	1.05 (103.37)	0.294	1	− 1.12 × 10^–5^ to 3.78 × 10^–5^	0.0404
Retrolenticular internal capsule	6.55 × 10^–5^	4.66 (102.67)	9.61 × 10^–6^	0.00013	3.81 × 10^–5^–9.28 × 10^–5^	0.168
Anterior corona radiata	6.15 × 10^–5^	4.58 (102.97)	1.32 × 10^–5^	0.000178	3.53 × 10^–5^–8.76 × 10^–5^	0.163
Superior corona radiata	5.62 × 10^–5^	4.41 (101.53)	2.6 × 10^–5^	0.00035	3.14 × 10^–5^–8.11 × 10^–5^	0.152
Posterior corona radiata	6.59 × 10^–5^	4.78 (100.87)	5.88 × 10^–6^	7.94 × 10^–5^	3.91 × 10^–5^–9.27 × 10^–5^	0.175
Posterior thalamic radiation	8.43 × 10^–5^	5.11 (107.40)	1.41 × 10^–6^	1.9 × 10^–5^	5.23 × 10^–5^–0.000116	0.197
Sagittal stratum	8.53 × 10^–5^	5.47 (102.99)	3.21 × 10^–7^	4.33 × 10^–6^	5.48 × 10^–5^–0.000116	0.219
External capsule	5.32 × 10^–5^	4.99 (102.85)	2.46 × 10^–6^	3.33 × 10^–5^	3.25 × 10^–5^–7.39 × 10^–5^	0.189
Cingulum—cingulate	1.84 × 10^–5^	1.4 (91.97)	0.164	1	− 7.39 × 10^–6^ to 4.46 × 10^–5^	0.0361
Cingulum—hippocampus	4.8 × 10^–5^	4.65 (105.13)	9.52 × 10^–6^	0.000129	2.8 × 10^–5^–6.8 × 10^–5^	0.169
Superior longitudinal fasc	6.33 × 10^–5^	5.01 (95.59)	2.52 × 10^–6^	3.4 × 10^–5^	3.87 × 10^–5^–8.78 × 10^–5^	0.185
Superior frontooccipital fasc	4.35 × 10^–5^	3.36 (99.08)	0.00112	0.0151	1.83 × 10^–5^–6.87 × 10^–5^	0.0936
Uncinate fasc	3.24 × 10^–5^	2.72 (108.98)	0.00769	0.104	9.05 × 10^–6^–5.55 × 10^–5^	0.0635
Tapetum	9.57 × 10^–5^	2.29 (96.30)	0.0243	0.327	1.45 × 10^–5^–0.000177	0.0453

**Table 5:** Group differences in radial diffusivity.

	β	t(df)	p_uncorrected_	p_FDR_	CI_99.99%_	R^2^
Cerebral WM	6.19 × 10^–5^	7.84 (108.27)	3.4 × 10^–12^	4.6 × 10^–11^	4.63 × 10^–5^–7.73 × 10^–5^	0.355
Cerebellar WM	3.57 × 10^–5^	4.55 (106.18)	1.41 × 10^–5^	0.00019	2.05 × 10^–5^–5.1 × 10^–5^	0.163
Corpus callosum—genu	0.000103	5.53 (103.04)	2.47 × 10^–7^	3.33 × 10^–6^	6.66 × 10^–5^–0.000139	0.222
Corpus callosum—body	7.74 × 10^–5^	6.59 (108.79)	1.65 × 10^–9^	2.23 × 10^–8^	5.39 × 10^–5^–0.0001	0.283
Corpus callosum—splenium	8.51 × 10^–5^	6.39 (108.46)	4.2 × 10^–9^	5.67 × 10^–8^	5.92 × 10^–5^–0.000111	0.275
Cerebral peduncle	3.46 × 10^–5^	1.97 (99.84)	0.0511	0.453	− 1.49 × 10^–7^ to 7.05 × 10^–5^	0.0426
Corticospinal TRACT	3.71 × 10^–5^	2.39 (92.83)	0.0187	0.168	6.96 × 10^–6^–6.73 × 10^–5^	0.0934
Medial lemniscus	0.000101	3.67 (102.18)	0.000393	0.0053	4.67 × 10^–5^–0.000155	0.104
Pontine crossing tract	6.64 × 10^–5^	5.07 (106.06)	1.68 × 10^–6^	2.27 × 10^–5^	4.09 × 10^–5^–9.18 × 10^–5^	0.194
Middle cerebellar peduncle	4.71 × 10^–5^	4.62 (109.00)	1.06 × 10^–5^	0.000143	2.73 × 10^–5^–6.69 × 10^–5^	0.161
Inferior cerebellar peduncle	5.16 × 10^–5^	3.29 (108.31)	0.00136	0.0183	2.09 × 10^–5^–8.26 × 10^–5^	0.0913
Superior cerebellar peduncle	7.91 × 10^–5^	2.88 (95.91)	0.0049	0.0662	2.49 × 10^–5^–0.000135	0.0719
Anterior limb internal capsule	3 × 10^–5^	4.93 (889.80)	9.6 × 10^–7^	1.3 × 10^–5^	1.82 × 10^–5^–4.19 × 10^–5^	0.173
Posterior limb internal capsule	2.38 × 10^–5^	3.45 (108.79)	0.000802	0.0108	1.04 × 10^–5^–3.72 × 10^–5^	0.0992
Retrolenticular internal capsule	4.6 × 10^–5^	6.08 (108.99)	1.8 × 10^–8^	2.42 × 10^–7^	3.1 × 10^–5^–6.07 × 10^–5^	0.253
Anterior corona radiata	7.63 × 10^–5^	7.64 (108.37)	9.17 × 10^–12^	1.24 × 10^–10^	5.68 × 10^–5^–9.57 × 10^–5^	0.352
Superior corona radiata	3.82 × 10^–5^	4.91 (107.09)	3.26 × 10^–6^	4.4 × 10^–5^	2.29 × 10^–5^–5.34 × 10^–5^	0.184
Posterior corona radiata	6.67 × 10^–5^	6.34 (108.96)	5.27 × 10^–9^	7.11 × 10^–8^	4.58 × 10^–5^–8.74 × 10^–5^	0.27
Posterior thalamic radiation	8.55 × 10^–5^	7.58 (108.31)	1.26 × 10^–11^	1.7 × 10^–10^	6.33 × 10^–5^–0.000108	0.348
Sagittal stratum	7.63 × 10^–5^	6.9 (108.64)	3.68 × 10^–10^	4.97 × 10^–9^	5.35 × 10^–5^–9.85 × 10^–5^	0.301
External capsule	4.71 × 10^–5^	7.93 (108.74)	2.09 × 10^–12^	2.83 × 10^–11^	3.54 × 10^–5^–5.88 × 10^–5^	0.368
Cingulum—cingulate	6.83 × 10^–5^	7.2 (106.41)	9.12 × 10^–11^	1.23 × 10^–9^	4.91 × 10^–5^–8.72 × 10^–5^	0.326
Cingulum—hippocampus	4.84 × 10^–5^	5.13 (105.46)	1.33 × 10^–6^	1.79 × 10^–5^	2.98 × 10^–5^–6.68 × 10^–5^	0.198
Superior longitudinal fasc	5.73 × 10^–5^	7.15 (108.61)	1.07 × 10^–10^	1.44 × 10^–9^	4.13 × 10^–5^–7.31 × 10^–5^	0.316
Superior frontooccipital fasc	2.8 × 10^–5^	3.41 (106.83)	0.000923	0.0125	1.2 × 10^–5^–4.39 × 10^–5^	0.098
Uncinate fasc	4.97 × 10^–5^	4.4 (102.63)	2.66 × 10^–5^	0.000359	2.65 × 10^–5^–7.23 × 10^–5^	0.153
Tapetum	0.000139	3.47 (94.04)	0.000795	0.0107	6.07 × 10^–5^–0.000218	0.101

### Relationships between WM microstructure and motor outcomes in DM1

**Figure 3:**
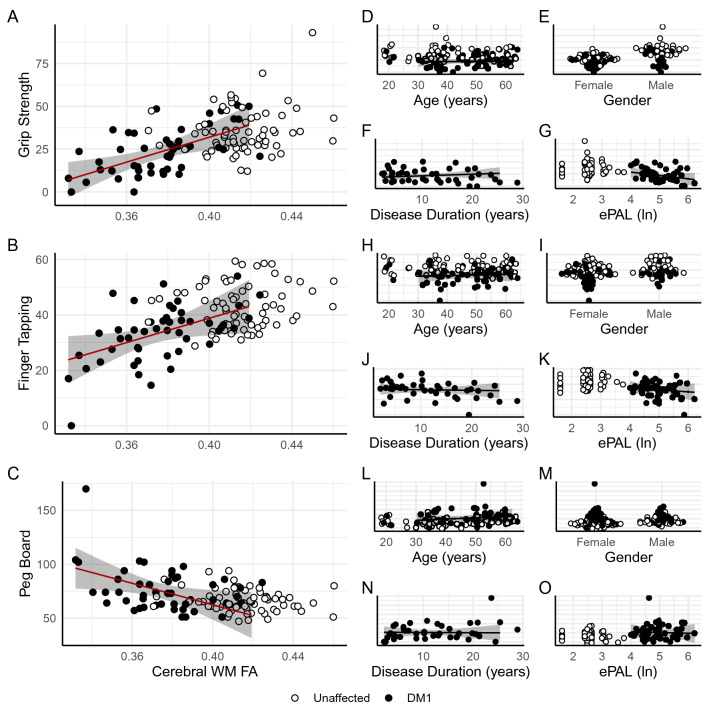
Cerebral white matter fractional anisotropy predicts motor outcomes. Cerebral WM FA predicts grip strength (panel **A**), finger-tapping (panel **B**), and peg board performance (panel **C**) in DM1. Other variables included in statistical models including age, sex, disease duration, and genetic burden are not significantly related motor outcomes (panels **D**–**O**). White circles indicate values for unaffected individuals for comparison, though are not included in statistical models. Lines represent fitted values from the respective models, red indicates significant effects, and the shaded regions represent estimates of the 95% confidence intervals. Data were plotted using R (version 3.5.0, https://www.r-project.org/)^25^.

**Figure 4:**
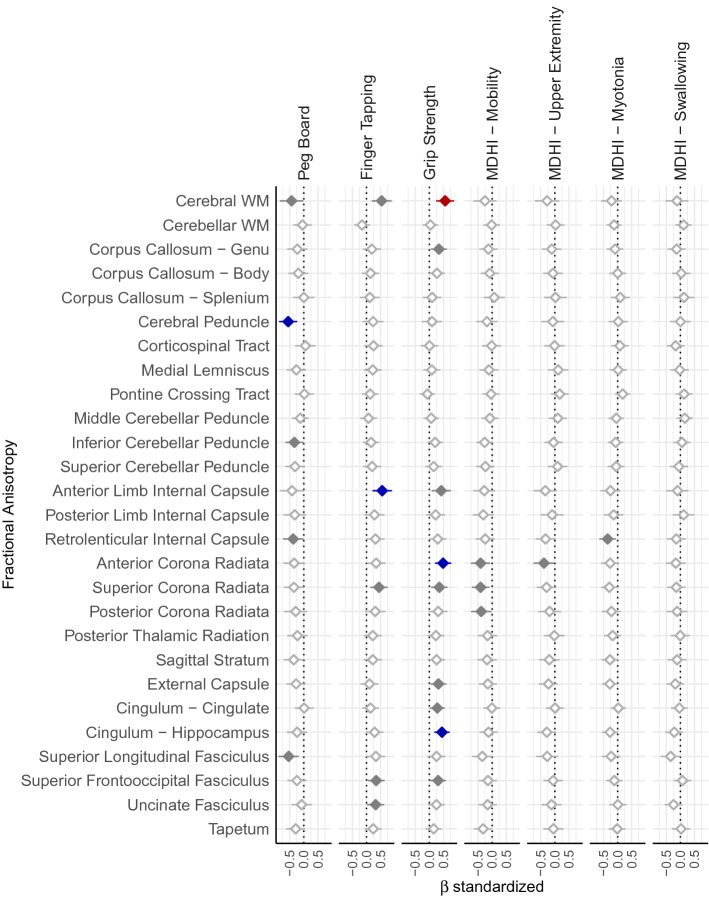
Fractional anisotropy predicting motor outcomes. The figure lists standardized betas listed on the x-axis for the association between regional FA (y-axis) and motor outcomes (top facets). Significant associations are marked by color, where red indicates VOIs where FDR-corrected *p* < 0.05, blue indicates FDR-corrected *p* < 0.1, gray indicates uncorrected *p* < 0.05, and white indicates uncorrected *p* > 0.05. Error bars represent the 95%. Data were plotted using R (version 3.5.0, https://www.r-project.org/)^25^.

**Figure 5:**
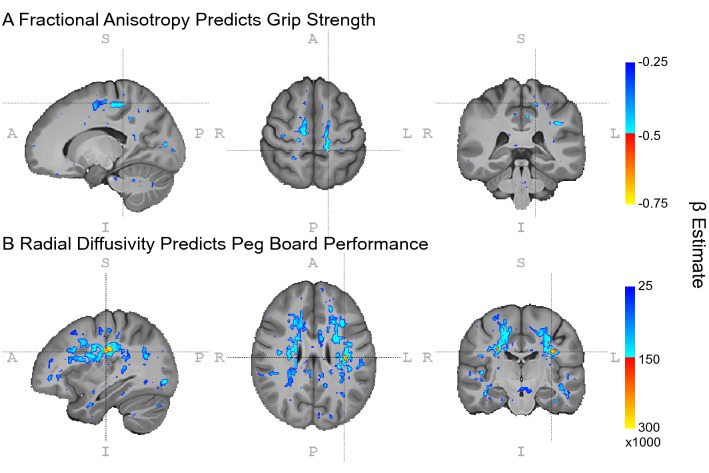
WM microstructure predicts motor outcomes. Fractional anisotropy predicts grip strength independently of disease duration and genetic burden. Voxelwise LME models suggest this relationship may be localized to WM underlying motor cortex (panel **A**). Radial diffusivity predicts performance on the peg board task, where voxelwise LME models suggest this relationship may be widespread throughout the brain (panel **B**). Cold colors indicate beta estimates for the group comparison in LME models thresholded at p_uncorrected_ < 0.05; hot colors indicate estimates thresholded at p_uncorrected_ < 0.01. Neuroimages were created using ITK-SNAP (version 3.8.0, http://www.itksnap.org/)^26^. Inkscape (version 0.1, https://inkscape.org/) was used to add labels and color bars to the figure.

WM integrity predicted several motor outcomes in LME models after controlling for fixed effects of disease duration, genetic burden, age, and sex and the random effect of family in DM1-affected participants only. Decreases in cerebral WM FA were related to decreases in grip strength, finger tapping, and peg board performance. The positive relationship between grip strength and overall fractional anisotropy in the cerebral cortex was significant (Fig. 3A, β = 329, CI_95%_ = 144–515, t_(39.00)_ = 3.48, *p* = 0.00125, p_FDR_ = 0.0336, R^2^ = 0.218). Similarly, there was a positive association between cerebral WM FA and finger tapping performance (Fig. 3B, β = 246, CI_95%_ = 85.8–406, t_(39.00)_ = 3.01, *p* = 0.00455, p_FDR_ = 0.123, R^2^ = 0.173) that approached significance, and a negative relationship between peg board performance and cerebral WM FA (Fig. 3C, β = − 377, CI_95%_ = − 741 to − 13.4, t_(38.36)_ = − 2.03, *p* = 0.0491, p_FDR_ = 0.663, R^2^ = 0.0859), see Fig. 3 for these specific models and Figs. 4 and 5 as well as Supplementary Data for complete results. To explore the validity and generalizability of these models, we conducted a cross-validation procedure using a jackknife (leave-one-out) resampling approach. For effects of cerebral WM FA on grip strength, finger tapping, and peg board performance, bias was low (− 0.007, − 0.009, − 0.021 standard deviations respectively) and all measures of normalized mean absolute error (nMAE) and root mean squared error (nRMSE) were less than 1 for both training and test sets. Moreover, the ratios of error measures between training and test sets was ~ 1 for all models, which suggests that our models are reasonably reliable when generalized to new data (see Supplementary Data for all cross-validation results). Critically, neither disease duration nor genetic burden were significant predictors of muscle/motor function independently of cerebral WM FA (all uncorrected *p*’s > 0.05). This is consistent with the notion that WM FA is a proximal mediator of muscle and motor deficits in DM1.

**Figure 6:**
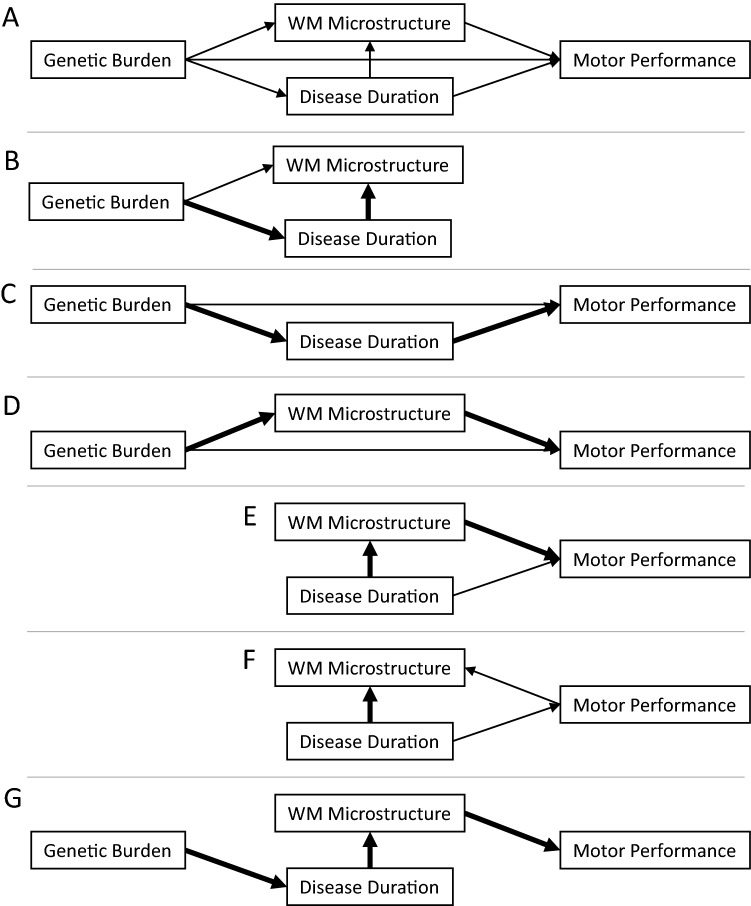
Causal mediation models. We explored a set of potential causal models using a mediation framework whereby we explore possible causal paths between genetic burden, disease duration, WM microstructure and Motor outcomes (Panel **A**). Each section of this pathway was analyzed, significant portions of each pathway are indicated by thick arrows, non-significant pathways are indicated by thin arrows (Panels **B**–**F**). Panel **G** represents the causal model indicated by mediation analyses of our data, where genetic burden is mediated by disease duration, disease duration is mediated by WM microstructure which in turn is the proximal cause of motor performance decline. Visualization was created using Inkscape software (version 0.1, https://inkscape.org/).

Our results are consistent with the notion that cerebral WM FA mediates between disease duration, genetic burden, and grip strength. It is possible that other ‘causal’ pathways remain (see Fig. 6A for a diagram of all causal pathways). For instance, genetic burden (see Fig. 6D) and disease duration (see Fig. 6E) may make independent contributions to motor outcomes or may be mediated by WM microstructure; the relationships between genetic burden and WM microstructure (see Fig. 6B) and motor outcomes (see Fig. 6C) may be mediated by disease duration given that earlier onsets are associated with longer CTG repeat lengths; and/or the direction of causality could potentially be inverted, where changes in motor abilities result in changes in cerebral WM (see Fig. 6F). We conducted a set of follow-up mediation analyses to explore how well each of these causal pathways match our data. According to the criteria for mediation^17^, we consider mediation pathways significant if: (1) there is a relationship between the predictor (e.g., genetic burden) and the outcome (e.g., grip strength), (2) there is a relationship between the mediator (e.g., cerebral WM FA) and the predictor, and (3) there is a relationship between the mediator and the outcome while controlling for the predictor. Furthermore, if (4) the predictor does not predict the outcome after considering the mediator a fully mediated “causal” pathway is indicated^17^.

First, mediation analyses suggest that ePAL is not the proximal cause of decreases in motor performance in DM1, as cerebral WM FA mediated the effect between ePAL and grip strength (Indirect Effect (δ) = − 6.59, CI_95%_ = − 16.12 to 0.09, *p* = 0.06; Direct Effect (ζ) = − 3.09, CI_95%_ = − 14.33 to 7.73, *p* = 0.62) but disease duration did not mediate this effect (δ = − 3.61, CI_95%_ = − 9.33 to 1.59, *p* = 0.22; ζ = − 5.23, CI_95%_ = − 16.88 to 7.98, *p* = 0.40). Second, disease duration mediated between ePAL and cerebral WM FA (δ = − 0.015, CI_95%_ = − 0.027 to 0, *p* ~ 0; ζ = − 0.01, CI_95%_ = − 0.03 to 0.01, *p* = 0.52). Third, cerebral WM FA mediated between disease duration and grip strength (δ = − 0.646, CI_95%_ = − 1.36 to − 0.21, *p* ~ 0; ζ = 0.24, CI_95%_ = − 0.442 to 0.87, *p* = 0.60). Together these results suggest that the underlying genetic cause of the disease (CTG repeat expansion) results in progressive changes (decreasing over time) in cerebral WM microstructure that in turn result in decreased motor performance (i.e., reduced grip strength). Moreover, mediation analysis in which the direction of causation in the model is reversed, i.e., to explore whether motor deficits mediate between disease duration and cerebral WM FA, we find only weak evidence of this mediation effect (δ = − 0.0003, CI_95%_ = − 0.0007 to 0, *p* = 0.06), while the direct path between disease duration and cerebral WM FA remains highly significant (ζ = − 0.0014, CI_95%_ = − 0.0022 to 0, *p* ~ 0). Our data are consistent with the notion that CTG repeat length modulates the age of onset of DM1, while the longer DM1-affected individuals progress with the disease the more cerebral WM degradation they experience, and this WM degradation leads to decline in motor performance (see Fig. 6G).

There is some evidence of regional contributions of WM FA to motor performance. Peg board performance was related to WM FA in the cerebral peduncles (β = − 263, CI_95%_ = − 416 to − 111, t_(19.32)_ = − 3.39, *p* = 0.00304, p_FDR_ = 0.0551, R^2^ = 0.141); finger tapping was related to WM FA in the anterior limb of the internal capsule (β = 157, CI_95%_ = 60.9–254, t_(39.00)_ = 3.2, *p* = 0.00274, p_FDR_ = 0.074, R^2^ = 0.191), and grip strength was related to WM FA in the anterior corona radiata (β = 170, CI_95%_ = 69.6–270, t_(39.00)_ = 3.32, *p* = 0.00197, p_FDR_ = 0.0531, R^2^ = 0.203) and hippocampal portion of the cingulum (β = 214, CI_95%_ = 85.4–343, t_(39.00)_ = 3.26, *p* = 0.00232, p_FDR_ = 0.0574, R^2^ = 0.197) (see Fig. 5A). Cross-validation demonstrates these models have low bias ( <|0.074| SD), error measures are less than 1 SD, and the ratio of error measures between training and test sets varies from 1.272 to 2.216. This suggests that regional measures are less reliable than more global measures of cerebral WM FA, which may in part be due to more individual variation in smaller brain regions compared to an individual average across many brain regions. WM FA did not tend to be a predictor of subjective ratings of motor impairment (all uncorrected *p*’s > 0.05).

Axial diffusivity did not tend to be a significant predictor of motor function, except for a trend where WM AD in the superior cerebellar peduncle predicted peg board performance (β = 4.55 × 10^4^, CI_95%_ = 1.81 × 10^4^–7.3 × 10^4^, t_(31.96)_ = 3.25, *p* = 0.00271, p_FDR_ = 0.0733, R^2^ = 0.163), see Supplementary Data for full results. Radial diffusivity in the external capsule significantly predicted peg board performance (β = 3.95 × 10^5^, CI_95%_ = 2.07 × 10^5^–5.84 × 10^5^, t_(35.10)_ = 4.11, *p* = 0.000228, p_FDR_ = 0.00616, R^2^ = 0.258) and there were trends where WM RD in the superior cerebellar peduncle (β = 6.27 × 10^4^, CI_95%_ = 2.33 × 10^4^–1.02 × 10^5^, t_(36.48)_ = 3.12, *p* = 0.00357, p_FDR_ = 0.0964, R^2^ = 0.173), sagittal stratum (β = 1.75 × 10^5^, CI_95%_ = 6.83 × 10^4^–2.81 × 10^5^, t_(36.92)_ = 3.22, *p* = 0.0027, p_FDR_ = 0.0729, R^2^ = 0.198) and hippocampal cingulum (β = 2.29 × 10^5^, CI_95%_ = 9.69 × 10^4^–3.61 × 10^5^, t_(19.30)_ = 3.4, *p* = 0.00297, p_FDR_ = 0.0801, R^2^ = 0.162) predicted peg board performance (see Fig. 5B). There was also a trend where RD in the external capsule predicted subjective ratings of mobility (β = 3.78 × 10^5^, CI_95%_ = 1.4 × 10^5^–6.16 × 10^5^, t_(37.00)_ = 3.12, *p* = 0.00353, p_FDR_ = 0.0954, R^2^ = 0.19), see Supplementary Data for full results. Cross-validation demonstrates these models have low bias ( <|0.08| SD), error measures are all less than 1 SD, and error ratios between training and test sets are close to 1, except for the relationship between external capsule RD and subjective ratings of mobility (~ 2). These results are consistent with the notion that averages across brain regions within individuals and measures that summarize other measures (i.e., FA is a more sensitive but less specific measure of WM integrity compared to AD and RD).

### Effects of disease duration and genetic burden on motor outcomes

While WM FA, AD and RD values did not tend to be significant predictors of subjective ratings of motor impairments, disease duration tended to be a non-significant predictor of subjective measures of motor impairments in mobility, myotonia, and swallowing, but not upper extremity function (all uncorrected *p*’s > 0.05). All non-significant effects were in the same direction, where longer disease durations were associated with higher subjective ratings of motor impairment. By contrast, genetic burden, while controlling for disease duration and WM microstructure did not predict motor impairments (all uncorrected *p*’s > 0.05).

### Neuropsychological and cognitive contributors to motor outcomes

Intellectual ability and neuropsychological impairments can potentially impact performance on motor tasks, e.g., grip strength and cognitive declines both accompany increasing age and are potentially but not necessarily causally related^18^. For example, deficits in motor performance may be manifestations of lack of attention to the task or lack of motivation to complete the task to the best of one’s ability. Likewise, mood disturbances may impact one’s ability to successfully engage with tasks, thus hindering performance. As a follow-up analysis, we explored whether neuropsychological measures predicted motor performance in participants with DM1. We limited this follow-up analysis to grip strength, finger-tapping, and peg board performance as these measures exhibited significant (or trending) relationships to cerebral WM FA. Furthermore, if the relationships between neuropsychological measures and motor outcomes were significant, we conducted a causal mediation analysis to explore whether cerebral WM FA mediated these relationships.

Grip strength was significantly related to full-scale IQ (β = 0.35, CI_95%_ = − 0.03 to 0.72, t_(36.00)_ = 2.12, *p* = 0.041) and this effect was indeed mediated by cerebral WM FA (δ = 0.41, CI_95%_ = 0.08–0.78, *p* = 0.02, ζ = 0.02, CI_95%_ = − 0.52 to 0.51, *p* = 0.9). These results are consistent with the notion that cerebral WM FA *fully mediates* the relationship between full-scale IQ and grip strength, according to the criteria for mediation^17^. All other neuropsychological measures were not significant predictors of grip strength.

Finger tapping was significantly predicted by full-scale IQ (β = 0.33, CI_95%_ = 0.04–0.66, t_(34.51)_ = 2.51, *p* = 0.017), COWA scores (β = 0.34, CI_95%_ = 0.02–0.66, t_(32.96)_ = 2.44, *p* = 0.02), and Trails B performance (β = − 0.15, CI_95%_ = − 0.29 to 0.01, t_(35.74)_ = − 2.66, *p* = 0.011). Moreover, these relationships were *fully mediated by cerebral WM FA*: full-scale IQ (δ = 0.24, CI_95%_ = 0.06–0.48, *p* = 0, ζ = 0.17, CI = − 0.08 to 0.47, *p* = 0.32), COWA (δ = 0.09, CI_95%_ = − 0.03 to 0.21, *p* = 0.08, ζ = 0.24, CI_95%_ = − 0.03 to 0.49, *p* = 0.1), and Trails B (δ = − 0.09, CI_95%_ = − 0.17 to − 0.03, *p* = 0.02, ζ = − 0.07, CI_95%_ = − 0.21 to 0.03, *p* = 0.24).

Peg board performance was significantly predicted by self-reported apathy (β = 1.25, CI_95%_ = 0.10–2.44, t_(36.00)_ = 2.62, *p* = 0.013), full-scale IQ (β = − 0.95, CI_95%_ = − 1.56 to − 0.37, t_(35.13)_ = − 3.69, *p* = 0.00076), Trails B performance (β = 0.39, CI_95%_ = 0.05–0.71, t_(35.31)_ = 3.38, *p* = 0.0018), judgement of line orientation performance (β = − 1.61, CI_95%_ = − 3.56 to 0.27, t_(33.00)_ = − 2.04, *p* = 0.049), Bender-Gestalt scores (β = − 1.03, CI_95%_ = − 1.87 to − 0.20, t_(30.37)_ = − 3.40, *p* = 0.0019), categories completed on the Wisconsin Card Sorting Task (β = − 4.78, CI_95%_ = − 11.19 to 1.17, t_(33.91)_ = − 2.40, *p* = 0.022), and score on Benton Faces (β = − 2.55, CI_95%_ = − 4.61 to − 0.61, t_(36.00)_ = − 3.45, *p* = 0.0014). However, in contrast to grip strength and finger tapping, cerebral WM FA did not mediate any of these effects, which is consistent with our observation of a weaker relationship between peg board performance and FA in general (all *p*’s for indirect effects > 0.12).

## Discussion

The current study replicates several other studies documenting the severity of cerebral white matter microstructural change in DM1. The study expands previous findings by showing white matter pathology was directly predictive of motor abnormalities such as grip strength, finger tapping, and peg board performance, even after controlling for factors such as disease duration and genetic burden (length of CTG repeat or ePAL). While there are primary genetic defects driving muscle pathology in DM1, our results support the notion that there may also be a role for brain pathology driving motor abnormalities.

Utilizing novel physiology measures of lower leg muscle function (soleus), our group recently showed that DM1 patients exhibited co-occurring alteration of spinal and trans-cortical reflex properties, providing strong support for the role of CNS abnormalities in muscle dysfunction in DM1^19^. Other neuroimaging studies have supported the notion that muscle impairment is linked to regional decreases in fractional anisotropy^12,13^. However, these studies have included either a relatively small number of participants with DM1 (N = 18)^12^ or have relied on a clinical rating scale (the Muscular Impairment Rating Scale [MIRS^20^])^13^ that captures motor impairment on an ordinal scale. The typical voxel-based procedures that have been used to explore relationships between FA and the MIRS^13^ assume that the numerical distance between adjacent categories is equal, which may or may not be accurate, thus results must be considered approximations. Our results support and extend the findings of this previous work, by recruiting a large sample of DM1 participants and using an enhanced statistical model that enabled us to account for whether disease duration and genetic burden are driving these effects. Taken together, these findings demonstrate that motor outcomes and WM integrity are co-varying, independent of the duration and genetic factors.

In DM1, muscle function may be affected by several factors: pure muscle weakness, myotonia (prolonged contraction/poor relaxation after contraction), or muscle fatigue. However, the amount of true muscle atrophy and the longitudinal progression over time in DM1 patients is not fully understood. A review of this disorder raised the question of DM1 being a model for premature aging, having some similarities to sarcopenia which may also have peripheral and central influences combined^21^. The observation that WM microstructure measures predict motor outcomes even after controlling for disease duration and genetic burden is consistent with the notion that WM degradation in DM1 mediates between the ultimate causes of the disease and motor symptoms. Indeed, our follow-up mediation analysis confirms that in our sample cerebral WM FA *completely mediates* between disease burden and grip strength (the direct path from genetic burden and disease duration was no longer significant after accounting for the indirect path where these effects were mediated by WM FA), supporting the notion that this relationship is potentially causal with white matter damage leading to motor dysfunction. Our data and mediation analyses are not consistent with the notion that there is a strong retrograde effect whereby muscle changes lead to alterations in cerebral WM microstructure. Further research should target this relationship in a longitudinal sample where changes in WM FA can be explored in the context of DM1 progression. This relationship between cerebral WM FA and grip strength could prove to be crucial for translational research where disease progression in the context of treatment regimens must be measured.

Our data are consistent with the notion that WM degradation in DM1 is systemic rather than regionally specific. Additional support for this notion is that WM degradation mediates the relationships between neuropsychological measures and motor outcomes. Specifically, our results are consistent with the notion that systemic WM decay results in cognitive impairments and contributes to motor decline. Importantly, WM degradation cannot be the only mechanism that is causing cognitive and muscle/motor decline. For example, our result whereby performance on the peg board task was not mediated by WM integrity suggests that there are likely other non-WM neurological factors impacted by DM1 that result in both cognitive and motor impairments. Despite most regions exhibiting some degree of degradation, the amount of degradation does vary regionally. Moreover, there appears to be at least some degree of localization of the relationship between WM FA decline and motor outcomes. For example, FA in the WM near the motor cortex predicts grip strength most strongly, peg board performance tended to be associated with WM measures that inputs and outputs for the cerebellum, and finger tapping with the anterior limb of the internal capsule near the basal ganglia. While these fluctuations in WM integrity likely relate to severity and variety of symptoms in a regionally specific way, aggregating FA values across the whole brain may provide a stronger, more consistent biomarker for translational research.

Despite the relationship between FA and muscle/motor impairments in DM1, the nature of deficits in WM structural integrity remains unclear and could be a fruitful topic for future research. Our results, specifically our observation that AD did not have any significant relationship with motor outcomes while RD did (albeit weaker than FA), may provide a hint of the underlying WM issues. These results are more consistent with the notion that while axons may not be damaged per se (no change in AD) there may be dys- or demyelination (increased RD)^22,23,for a review see: 24^. Further research that includes histological examination of WM microstructure in DM1 could address the source of these deficits. This work may be limited by the sample size, where a larger sample may elucidate more specific changes in WM measures of AD and RD, which may point to specific WM pathologies.

We did not observe a relationship between patient-reported, subjective motor symptoms as measured by the MDHI and WM microstructure. These subjective reports may be confounded by patient apathy and/or insight. However, disease duration tended to be weakly related to self-reported myotonia, swallowing, and mobility. We speculate that WM degradation may go unnoticed in terms of symptomatology until some critical threshold is met; particularly considering our sample of patients with DM1 who have relatively mild symptoms. Further research, in a longitudinal setting would allow us to elucidate the time course of WM degradation and the patient experience of symptoms and establish the natural history of FA abnormalities and muscle pathology.

This work is potentially limited by sample size and its cross-sectional nature, as discussed above. Increased sample size and repeated measures over time would allow us to explore in more detail the underlying WM pathology (e.g., larger sample would give more power to AD and RD results) and a longitudinal approach would provide further evidence for mediation if motor and WM degradation occur in concert. Furthermore, this work is limited due to the nature of MRI studies where more scanning time could yield higher resolution images which could yield better insight into specific WM pathology as well as possible spatial specificity within the brain. Finally, there may be other factors that contribute and/or mediate these effects that were not measured as a part of our study; with further research these unknown factors may come to light.

Given DM1 is a single gene disorder, there is a frenetic push towards development of gene knock-down and other therapies designed to treat or slow down disease progression. One important caveat is that although some therapies have been developed for delivery to muscle, our results are consistent with the notion that complimentary treatments delivered to the brain could also benefit motor function. Our results suggest that cerebral WM integrity may be a critical tool in evaluating these therapies.

## Methods

### Participants

The Iowa Brain DM1 study focuses on recruitment of individuals with adult-onset DM1 (those who exhibited disease-related symptoms after the age of 21 years old) as well as individuals who are at-risk for DM1 (those with a family history of DM1), but have not yet been genetically tested. Participants were recruited from our own multidisciplinary specialty clinic for DM1 at the University of Iowa and through the Myotonic Dystrophy Foundation. A control group consisting of healthy adults was recruited from partners of DM1 participants, and from the Iowa City area via advertisements. Exclusion criteria for all participants included: MRI contraindication, a history of serious head injury, or a chronic neurological disorder other than DM1. Healthy adults were additionally required to be without a history of substance abuse, psychiatric disease, or major medical disease, including: heart disease, sleep disorder, vascular disease, uncontrolled hypertension, cancer, diabetes mellitus, lung disease, and autoimmune conditions. The current sample included 119 individuals: 61 healthy adults, 45 individuals with confirmed DM1 and 13 individuals with a family history of DM1 who had not undergone confirmative testing. Participants underwent genetic testing for research purposes only. At-risk individuals who were determined to have CTG repeat length ≥ 50 were included in the DM1 group (N = 5); the remainder had CTG repeat length in the non-expanded range and were included in the group of healthy adults (N = 8). The final sample included 50 individuals with DM1 and 69 healthy adults. For the patients with DM1, disease duration was determined by the time at which they received a clinical diagnosis of DM1. This ranged from 0 (those who were at-risk, found to have the gene-expansion but no clinical symptoms) to 28.9 years with a mean of 8.88 years.

Research staff, clinicians, and scientists involved in this study remained blind to the genetic status of at-risk individuals. All data were de-identified, and all participants consented to non-disclosure of genetic results obtained as part of the study. All participants gave written, informed consent prior to enrolling in the protocol in accordance with the Declaration of Helsinki. The study was approved by the University of Iowa Institutional Review Board. All methods were performed in accordance with these guidelines and regulations.

### Estimated progenitor allele length (ePAL)

Genotyping of CTG repeat in DM1-affected participants, and at-risk individuals was completed by SP-PCR^27^. For each patient, four reactions were completed, each using a 300 pg genomic DNA template derived from blood leukocytes. CTG repeat lengths were estimated by comparison against DNA fragments of known length and molecular weight markers, using CLIQS software (TotalLabs UK Ltd.). The lower boundary of the expanded molecules in SP-PCR was used to estimate the progenitor (inherited) allele length (ePAL)^28^. ePAL is a major determinant of age at symptom onset^29^.

Estimation of CTG repeat length of the non-disease-causing allele(s) at-risk individuals and controls was determined by Illumina MiSeq sequencing, essentially as described for Huntington disease^30^. Barcoded primers that contained all the sequences required for MiSeq sequencing, combined with gene-specific sequences flanking the CTG repeats, were used to generate the amplicon sequencing library. Open-source bioinformatic tools on the Galaxy platform^31^ were used to process sequence reads and align them against custom reference sequences comprising unique DM1-specific flanking sequences, separated by 0 to 100 CTG repeats. The reference sequence(s) with the highest number of aligned reads corresponded to the number of CTG repeats in the non-disease-causing allele(s).

### Motor/muscle function

Fine motor skills were measured with the Lafayette Instruments finger tapping test and the grooved Pegboard. The finger tapping apparatus consists of a tapping key with a device for recording the number of taps. Participants completed five consecutive tap trials that were 10 s each. The dependent variable included the average number of taps across five trials using the dominant hand. The Grooved Pegboard test requires participants to insert keyed pegs into slots. The outcome measure of interest was time to completion in seconds using the dominant hand.

The Lafayette Instruments dynamometer was used to assess grip force in kilogram-force (kgf). While standing, participants squeezed the instrument as hard as they could six times (three trials using the dominant hand, three trials using the non-dominant hand). The dependent variable represents the average grip force of the dominant hand across three trials.

### Patient-reported motor/muscle function

The Myotonic Dystrophy Health Index (MDHI) is a disease-specific patient-reported outcome measure for myotonic dystrophy type-1^15,32^. It is composed of 114 items broken down within 16 individual subscales that together measure multi-factorial patient-reported burden of disease. Each item is rated on a 6-point Likert scale as to how much the item “impacts the participant’s life now.” The range of response options are “I don’t experience this” to “It affects my life severely.” Given the current emphasis on motor and muscle related outcomes, we utilized scores on only the relevant subscales, including: myotonia, mobility, upper extremity function, and swallowing.

### Magnetic resonance imaging

Participants who participated before June 2016 (N = 49, 24 unaffected, 25 with DM1) were scanned using a 3 T Siemens TrioTIM scanner (Siemens AG, Munich, Germany; 12 channel head coil, software version: syngo B17). Those who participated after June 2016 were scanned using a 3 T General Electric Discovery MR750w scanner (GE Medical Systems, Chicago, Il, 16 channel head and neck coil, software versions: 25.0, 25.1, and 26.0) (N = 65, 43 unaffected, and 22 with DM1). Participants completed DWI acquisitions with either a single-shell (B1000, 64 directions), multi-shell (B1000 and B2000, 29–30 directions per shell), or both (details provided in ***eTables 1 and 1 in the Supplement). Diffusion-weighted images were collected using echo planar recovery magnitude sequences collected in the axial plane. Anatomical T1-weighted and T2-weighted images were collected and used for co-registration, normalization, and labelling purposes using acquisition parameters described previously^33^.

### White matter FA

Diffusion-weighted images were processed using standard procedures of the FMRIB Diffusion toolbox from the FSL software package (http://www.fmrib.ox.ac.uk/fsl), where phase encoding distortion and eddy current artifacts were removed using topup and eddy tools respectively^34,35^. Following correction, diffusion tensor models were generated using dtifit, and from these tensors, scalar measures, including fractional anisotropy (FA), axial diffusivity (AD) and radial diffusivity (RD), were calculated. B0 maps were co-registered to T2-weighted images for each subject, which were in turn registered to their T1-weighted images, which were normalized to a standard space. All registrations consisted of rigid, affine, and nonlinear (symmetric normalization) components and were conducted using Advanced Normalization Tools^36^. All steps in the registration sequence were combined into a single transform as necessary and applied simultaneously to scalar maps (FA, AD, RD) to avoid compounding interpolation errors. Data were normalized to an unbiased average of the brains from the Human Connectome Project^37^, which itself was normalized to ICBM 2009b Nonlinear Asymmetric space^38^.

Regions of interest were extracted using the BRAINSAutoWorkup pipeline which optimizes tissue classification through an iterative framework and produces robust parcellation of brain regions results in a multi-scanner setting^39^. BRAINSAutoWorkup labels brain regions using a multi-atlas, similarity-weighted, majority-vote procedure (joint label fusion^40^) using a set of expert-segmented templates adapted from the Desikan-Killiany atlas^41^. White matter labels corresponding to cerebral and cerebellar regions were combined to generate regions of interest for each participant’s brain in its native space. For voxel-wise modelling and the JHU WM atlas^42–44^, spatially normalized scalar values were used.

### Statistical analyses

Statistics and figures (Figs. 1, 3, 4; not including neuroimaging) were generated using R 3.5.0^25^. Neuroimaging figures (Figs. 2, 5) were created using ITKSnap^26^ for brain overlays and Inkscape (version 0.1, https://inkscape.org/) for panel layout and generation of colour bars.

Demographic characteristics of the DM1 group and the unaffected group were summarized and compared by chi-squared test for proportions, 2-sample t-test for means, or Wilcoxon’s rank-sum tests for medians.

First, batch effects associated with scanner vendor and software version were harmonized using ComBat harmonization^45,46^ implemented in R. Group differences in functional outcomes and WM scalar values were examined using a linear mixed effects (LME) framework, where the random effect of family (to control for the effects of familial relationships among some individuals in our sample) and fixed effects of group, age, and sex were included in each model. Given the number of tests, models including functional outcomes and JHU regions were FDR-corrected using the Benjamini & Hochberg procedure^47^. LME models exploring WM values were also applied voxelwise to explore potential regionally specific WM degradation using tools implemented in R.

Second, the LME framework was used to assess whether WM microstructure, disease duration, and/or genetic burden predicted functional outcomes in DM1 participants only. Family identifiers were included as random effects and WM measures, disease duration (years from diagnosis), genetic burden (natural log transformed ePAL), age, and gender were included as fixed factors. Critically, measures of WM microstructure (fractional anisotropy, axial diffusivity, and radial diffusivity), disease duration, and ePAL were included in each model, such that the effect of each of these variables could be considered while controlling for the potential effects of the others. Interaction terms were not included in LME models, as we are interested in exploring *independent* effects of each of these variables rather than *conditional* effects (or the effect of one variable on another). P-values were FDR-corrected^47^. To visually explore regional variation in these relationships we ran these LME models voxel wise in addition to volume-of-interest-based measures. Given the exploratory goal of voxelwise models, these values were not FDR-corrected; rather a threshold of *p* < 0.001 and a cluster size of 25 contiguous voxels was used for visualization purposes.

### Ethical approval

All methods were performed in accordance with the guidelines and regulations of the Declaration of Helsinki and the University of Iowa Institutional Review Board.

## Supplementary Information

Supplementary Legends.

Supplementary Table 1.

Supplementary Table 2.
