# Supplementary material for: White matter microstructure relates to motor outcomes in myotonic dystrophy type 1 independently of disease duration and genetic burden
Source: Sci Rep. 2021 Mar 1;11:4886. doi: 10.1038/s41598-021-84520-2 (PMC7921687; doi:10.1038/s41598-021-84520-2)
Supplement: Supplementary file 1 — Supplementary Legends. [file 41598_2021_84520_MOESM1_ESM.docx]

Supplementary Information

White matter microstructure relates to motor outcomes in myotonic dystrophy type 1 independently of disease duration and genetic burden.

Timothy R. Koscik, PhD^*†1^(ORCID: 0000-0002-2551-8127); Ellen van der Plas PhD^†1^ (ORCID: 0000-0002-7490-6636); Laurie Gutmann, MD^2^, Sarah A. Cumming, PhD^3^ (ORCID: 0000-0002-0201-3660); Darren G. Monckton, PhD^3^; Vincent Magnotta, PhD^4^; Richard K. Shields, PhD^5^, Peggy C. Nopoulos, MD^1,2,6^

^†^Co-First Authors, Contributed Equally

^1^Department of Psychiatry, Carver College of Medicine, University of Iowa

^2^Department of Neurology, Carver College of Medicine, University of Iowa

^3^Institute of Molecular, Cell and Systems Biology, University of Glasgow

^4^Department of Radiology, Carver College of Medicine, University of Iowa

^5^Department of Physical Therapy and Rehabilitation Science, Carver College of Medicine, University of Iowa

^6^Department of Pediatrics, Carver College of Medicine, University of Iowa

##

## *Corresponding Author:

Timothy R. Koscik; 200 Hawkins Drive, Iowa City, IA, 52242; 319-384-6884 [timothy-koscik@uiowa.edu](mailto:timothy-koscik@uiowa.edu)

Supplementary Table 1

The included dataset “DM1_motor-WM-diseaseDuration-geneticBurden.tsv” is a tab-delimited file containing results of statistical modelling of all combinations of variables exploring relationships between WM micostructure (FA, AD, RD) and motor outcomes.

Supplementary Table 2

The included dataset “DM1_crossValidation.csv” is a comma-delimited file containing results of cross validation when modelling relationships between WM microstructure and motor outcomes in DM1.
